# Taking heart rate variability to the next level in sports: towards a multi-signal integration

**DOI:** 10.3389/fspor.2026.1720495

**Published:** 2026-01-22

**Authors:** Nicolas Bourdillon, Grégoire P. Millet

**Affiliations:** Institute of Sport Sciences, University of Lausanne, Lausanne, Switzerland

**Keywords:** accelerometer, baroreflex, fatigue, heart rate variability, photoplethysmography, respiratory sinus arrythmia

## Abstract

Heart rate variability (HRV) is most often used as a standalone, without integration of complementary data. Yet these data are at our fingertips, and a revolution might be just around the corner. This perspective article gives insight on how integrating accelerometery and continuous blood pressure and extracting ventilatory variables may improve the use of HRV, taking it to a whole new level for elite and recreational sports, assessing fatigue, and intensity domains. Also, the use of mass data could lead to seamless measurements as precise and relevant as in elite sports, which could be made available to the public for health assessment and follow-up.

## Introduction

Since immemorial times, athletes and coaches have sought to improve performance using elaborated training plans, which window frames span from a few weeks to four years or an entire elite sport career. The basic principle underlying this expected improvement in performance resides in accurately balancing overload and recovery periods to elicit positive physiological and psychological adaptations (1). However, this optimal balance is difficult to determine and challenging to reach, notably because it is volatile (intra- and inter-variability in athletes), and because both the external and the internal loads are complex. One of the most widespread physiological indicators is heart rate variability (HRV), it is easily accessible and holds vital information on the autonomic balance (2). The latter is commonly interpreted as sympathetic and parasympathetic influences on the heart, each being either complementary (both are required to be high to elicit performance improvement) or opposed (concomitant increase in sympathetic and decrease in parasympathetic is expected in response to a stressor, and vice versa during recovery). This harmonious yet antinomic relationship can be difficult to interpret in the context of training planification and performance in elite sports, and has been the topic of extensive literature and intense discussion (3–6).

Beyond this classic view of the autonomic balance, HRV holds information on two vital regulatory loops which are respiratory sinus arrythmia (RSA) and arterial baroreflex (BR). The former arises from the synchronisation between the respiratory movements and the heart so that instantaneous heart rate increases during inspiration and decreases during expiration (7). The latter arises from beat-to-beat blood pressure regulation where a decrease in blood pressure triggers an increase in heart rate and vice versa (8). These two regulatory loops are permanently interacting, inhibiting each other and influencing the heart beat-to-beat intervals (RR intervals).

HRV holds key information on the autonomic processes underlying health and physical performance, yet the way it is used by athletes and coaches is—in our view—not yet optimal. The only common ground between all the HRV methods and interpretations lies in recording a sequence of inter-heartbeat time intervals extracted either from an electrocardiogram (ECG), usually integrated in a chest strap, or from a photoplethysmogram (PPG) integrated in a wrist-worn device. The inter-beat intervals are simply the time elapsed between two consecutive heartbeats, taken at each R-wave in the ECG, constituting a RR-interval time series. The peak-to-peak intervals are extracted from the photoplethysmogram and used as a surrogate for RR intervals, which is trustable at rest in steady position e.g., during sleep (9), but not during intense exercise (10, 11). This RR-interval signal is pseudo periodic, which means that the physiological information of interest lies in its constituting waves. Therefore, the aim of any HRV analysis is to characterize these waves using dedicated computations either in the time-, frequency- or non-linear domains (2). In the everyday practice of sport science recording durations range from a few seconds to a full night and interpretations range from “just one more indicator” to “dictating the content of the next training session”; generally overlooking the importance of RSA and BR underlying the measured HRV.

The goal of this perspective article is to 1) summarize each current measurement method and their implications; and 2) envision the integration of complementary data and how it could bring innovation in the field.

## Measurement methods and their implications

### The ultra-short-term HRV (UST-HRV)

Ultra short term HRV are usually defined as 30-s or 1-min recordings, such short durations are very convenient for the athletes or coaches, can be performed on a (multi-) daily basis or can even be extracted from a continuous 24-h recording (12) on periods where the athlete is calm, relaxed and in a known given position (e.g., seated, supine or standing). The few HRV parameters computed are generally mean heart rate (HR), root mean square of the successive differences (RMSSD) and standard deviation (SD). Out of these parameters, RMSSD is clearly a parasympathetic indicator whilst SD is usually taken as a mix of sympathetic and parasympathetic influences or is used to derive a sympathetic indicator (13).

However, the periods of the waves most characteristic of the sympathetic nervous system (SNS) in RR recordings range between 7 and 25 s (2). In biomedical signal processing, 10 waveforms are generally needed to properly analyse a given wave, therefore catching a 25-s period may require at least 250 s (∼4 min) of recording (14). The assessment of the autonomic balance seems limited using UST-HRV as parasympathetic information is likely valid, but the sympathetic information must be taken cautiously. Also, pushing the interpretation to RSA or BR evaluation seems hazardous using such short recording durations, and in the absence of any complementary signal.

### The active orthostatic test

For this test, the recording of the RR intervals is performed in the supine followed by the standing position, generally for at least five minutes in each position. Nonetheless such measurement duration allows proper analysis of the longest sought waves (25-s period), but it also allows the analysis of two relevant body positions. In the supine position, RSA is expected to be the main influence on the RR-interval variability, of which both RMSSD and power in the high-frequency band (HF) seem good indicators, whilst BR should be almost non-existent as all organs are about the same height. In the standing position, the orthostatic stress decreases the influence of RSA on RR-intervals, to the benefits of the BR. The latter becomes the main influence on RR-interval variability, compared with the supine position. Also, the analysis of the few seconds during the postural shift from supine to standing brings highly relevant information since this period is strongly influenced by the quality of the sympathetic activation.

Such interpretation have been successfully used in elite athletes for the determination of overreaching (15, 16), the identification of different fatigue profiles (17) which have subsequently been used for training planification (18), correlated to World class podiums (19) and successfully used to better prescribe training content; e.g., the so-call HRV-guided training (20, 21). Yet, to allow for such accuracy, reproducibility is key. HRV recordings must be conducted in a very controlled environment typically after wake-up, whilst fasting, with an empty bladder and in a calm environment (2). Such constraints make daily measurements tough to perform over a prolonged period.

### The overnight measurement

Unlike the orthostatic test, the only constraint of this kind of measurement is to wear the recording device overnight. However, the interpretation of data is more cumbersome as the recordings are long and sleep stage/arousal dependant. The behaviour of HRV indices overnight remains to be clarified both in recreational athletes (22) and in elite athletes (23). Still, overnight HRV hold key information on sleep quality, which is essential for recovery, and RSA is most likely possible to assess given the long duration of these measurements. However, specific literature on these aspects in athletes seems missing and future research is required.

### Heart rate variability derived thresholds

All previous measurements were performed at rest or during sleep, yet in the context of sports science, HRV measurements are also relevant during exercise. One of the key goals of an incremental exercise to exhaustion is to determine the intensity domains, moderate below the first ventilatory threshold, heavy in-between the first and second ventilatory thresholds and severe beyond the second ventilatory threshold (24). These intensity domains are a key element of training prescription, yet their determination requires a cumbersome and costly equipment limiting their use (25). Performing an incremental exercise with just a chest strap may allow to accurately determine the intensity domains using HRV-derived threshold (5). Beyond HRV, the respiratory frequency can be efficiently derived from an ECG signal (26) embarked on a chest strap which, combined to HRV-derived thresholds, may yield accurate intensity domain determination. Additionally, other metrics characterizing endurance or exercise durability could be extracted and hold valuable information (27). Once again, the recording devices and data are in our hands, but effective signal processing and data integration is missing to reach satisfying accuracy.

### Hear rate recovery (HRR)

Last but not least, HRV can be analysed during post-exercise recovery, which goal is to assess parasympathetic reactivation (28, 29); the metrics and recording durations are quite well described in the literature (30, 31). In short, recording the RR intervals up to 10 min immediately following a given exercise can bring insightful information on parasympathetic reactivation and therefore on the quality of recovery. Again, HRR is not often seen in sport science studies and is non-existent in everyday practice, whilst it could easily be automatized, the required data being easily available. Alternative methods using only HRR for 5 min have been proposed and are widely used particularly in team sports (32).

### Quality of ECG

Most of the measurement conditions mentioned above imply rest or recovery during which motion artefacts are scarce, easily identified, corrected and/or removed before data analysis. However, obtaining an accurate RR time series during exercise is more challenging as the R-peak voltage needs to be high enough to be distinct from noise. Thus if there is too much noise measurement agreement to standard will not be satisfactory. Most recent textile, electrical probes and sensors must thrive to keep improving signal quality and expand the possibilities of subsequent signal processing.

## Taking HRV to the next level

### Accelerometery data

As described above, HRV is most often used as a standalone, without any complementary data, and its interpretation is usually limited to a vague sympathovagal balance (33), suddenly forgetting the underlying, yet key, RSA and BR mechanisms. Nowadays most HRV sensors record simultaneously the RR intervals and three-dimensional accelerometery data. An accelerometer worn on a chest strap allows to measure thoracic respiratory movements which result in a wave from which respiration can be isolated (34). Other options for extracting respiration from common chest straps include ECG-derived respiration (35), chest bio-impedance (36) and constraint gauge (37). However, extracting respiratory rate solely from RR-interval (RSA based) is less accurate and less trustable than the precited methods. Quantifying the presence and phase shift of this respiratory wave within the RR-interval signal allows the assessment of RSA and is routinely done in sleep studies (38) but is rarely done in sports science despite the data being at our fingertips (26). In addition, the most recent chest straps estimate the true lung volume, breath-by-breath, giving the possibility to estimate RSA even more accurately.

### Continuous blood pressure data

Recent technological improvements allowed wrist-worn device to measure continuous photoplethysmography signal and estimate continuous blood pressure trace (39). From such traces, baroreflex sensitivity can be assessed using well-established methods such as the sequence method or the standard deviation ratio method (40). Also, synchronizing an electrocardiogram and a continuous blood pressure trace allows to assess the central (heart) vs. peripheral (blood vessels) impact on specific circulatory parameters such as pulse wave velocity. This information allows to refine the assessment of the autonomic balance by having peripheral information in addition to the central information given by HRV, RSA and BR. The peripheral information may even be more sensitive than the central one in fatigue detection (41). However, wrist-worn devices are very sensitive to movement artefacts and to date blood pressure assessment or RR-interval estimation from such devices may only be valid at rest or during sleep (9) and not during intense exercise (42, 43).

### Next steps

At first, synchronized RR intervals, accelerometery and continuous blood pressure are needed, in the context of the controlled recording protocols described earlier in this article (e.g., orthostatic test). The existing algorithms of biomedical signal processing may be used to setup the basis of this data integration. Very quickly mass data could be gathered and Machine Learning approaches used to achieve the goal of having seamless recording, i.e., continuous recording from a wearable without the need for a controlled recording protocol, with the same accuracy as what is currently done using the controlled recording protocols in elite athletes. In other words, the accuracy and benefits of HRV-combined data used by elite coaches and athletes could be made available for the public, with all the health and prevention benefits that shall come along. However, seamless continuous monitoring is not synonymous with recording and analysing data in all conditions throughout the day. Such approach will likely require automatic recognition of time periods where the signals could be steady enough to be properly analysed. For valuable and accurate information, these periods will likely be during sleep, at rest or light exercise, but likely not during intense exercise or when movement or other confounding factors overload the signals.

## Discussion

### Integrating data

Bringing together and integrating HRV, RSA, BR and peripheral assessments by combining HRV, accelerometery and blood pressure trace could take the analysis of the autonomic nervous system to a new level. Literally, a revolution might be just around the corner as these data are easily acquired by wearable sensors, so that automatization could be performed, algorithms elaborated, and the information made available for anyone wearing such devices, all this in a few months/years' time. The capability to achieve HRV-integrated measurements way more relevant and specific than what is currently done is in our hands, but why does it matter and what could the benefits be in practice?

Commercially available devices already partially implement data integration and provide the users with a “readiness score” or a “blood battery” estimation comprising for example RMSSD, body temperature, sleep quality, and daily load. These scores are sometimes accompanied by very generic (i.e., non-specific) recommendations if any. Thus, except in the hands of a few experts in elite sports, the recommendations following HRV measurements are impact-less and largely ignored by the public. By integrating HRV and complementary data, the identification of the various fatigue profiles already described in the literature (17) could be automatized, and new fatigue profiles potentially identified.

Identifying a fatigue profile is drastically different from a score as the latter is generally only quantifying parasympathetic modulation to the heart, overlooking any other function of the human body. The assessment of central vs. peripheral factors, and the assessment of both RSA and BR feedback loops potentially leads to recommendations for the athlete that are specific, accurate, relevant and allow to recover quickly and efficiently from a given fatigue profile to a beneficial state, thereby contributing to optimize the overload/recovery balance. The recommendations would encompass training volume and intensity of course, but also other recommendations influencing the sympathovagal balance, e.g., cardiac coherence (44), massage (45), hot/cold bath (46), diet (47) etc.

In summary, during rest integrating HRV and chest-strap accelerometery allows to assess RSA, integrating HRV and continuous blood pressure allows to assess BR, integrating HRV and PPG allows to assess the peripheral vs. central determinants of the sympathovagal balance. During exercise integrating HRV, chest strap ECG-derived and accelerometery allows to accurately determine the intensity domains (5). Yet, there are several biases on HRV-derived thresholds compared with standard methods (e.g., ventilatory or lactate). seems to be on an individual level. However, taken together, HRVTs can be a promising alternative for prescribing exercise intensity in healthy, male athletes undertaking endurance activities but due to the heterogeneity of study design, threshold concepts, standardization, and lack of female participants, further research is necessary to draw more robust and nuanced conclusions (48).

All these integrations taken together could greatly improve the characterization of each athlete and allow very individualized and relevant recommendations regarding training and recovery prescriptions. The future of HRV integrated data seems bright, yet we can dream for even better.

### Recreational sports and general public

Beyond the world of elite sports, there are recreational sports and the general population. These two additional populations may be more reluctant than elite sports in performing rigorous well-controlled recordings, yet they wish to access the same relevance and accuracy as elite sports. The use of mass data would allow to perform data integration without the need of a controlled recording protocol and to automatize the association between each fatigue profile and its subsequently associated set of recommendations without the expertise of an elite coach. This means that with seamless measurements anyone could benefit a fatigue management system where specific and personalized recommendations could be updated daily. Such system would also allow to measure the benefits of the actions taken. The range of possible actions is wide and an exhaustive listing all the fatigue profiles and their corresponding recommended actions is beyond the purpose of the present perspective article. Yet, here is a glimpse of what the future could resemble: for example a fatigue profile showing good RSA and HF power but altered BR associated with off-balanced LF power could lead to recommend a training session oriented toward high-intensity repeated sprints, relatively short, possibly associated with alternated hot and cold baths in an attempt to stimulate the sympathetic nervous system without eliciting fatigue. Another example could be a global decrease in both RSA and BR functions associated with low or normal HRV, which could involve low-intensity continuous training sessions, associated with cardiac coherence, specific diet and increased sleep time to promote recovery.

Keeping on advancing into the future, sufficient data and knowledge could allow to anticipate the fatigue outbreaks, by adjusting the training/recovery either to trigger a given fatigue profile at a given moment (and optimize recovery and the subsequent beneficial effects on performance) or prevent the occurrence of an undesired fatigue profile negatively affecting performance. If such technology were available, prevention of performance decrement and injury in sports could be drastically reduced to the benefits of the athletes' health and cut down the subsequent financial burden. For the general population, burnout, orthostatic hypotension, acute fatigue and low energy levels for example may benefit from such research initiated in elite sport. The ability to anticipate burnout may benefit the health of millions of people and relieve the overwhelmed public health systems worldwide. Finally, arrythmia detection (49) may also contribute to improving athletes' and general public's health and precocious detection of cardiovascular risk. However, such clinical implementation with potential medical implications is beyond the scope of the present article and necessitates further research.

## Conclusion

HRV has already a significant impact in elite sports, yet, despite partial data integration, it is sometimes used as a standalone, which limits its usefulness. By integrating at least accelerometery and blood pressure data, methodological advances are just around the corner, bringing specific information on respiratory sinus arrythmia and arterial baroreflex, which are two vital feedback loops. Additionally, the translation of knowledge developed in elite sport to everyone may enable health preventive actions, patients monitoring and benefit recreational athletes.

## Data Availability

The original contributions presented in the study are included in the article, further inquiries can be directed to the corresponding author.
